# Biocompatible zwitterionic copolymer-stabilized magnetite nanoparticles: a simple one-pot synthesis, antifouling properties and biomagnetic separation†

**DOI:** 10.1039/c8ra06887a

**Published:** 2018-11-05

**Authors:** Supannika Boonjamnian, Thanida Trakulsujaritchok, Klaokwan Srisook, Voravee P. Hoven, Piyaporn Na Nongkhai

**Affiliations:** Sensor Innovation Research Unit (SIRU), Burapha University Muang Chonburi 20131 Thailand piyapornn@buu.ac.th; Department of Chemistry, Faculty of Science, Burapha University Muang Chonburi 20131 Thailand; Center of Excellence for Innovation in Chemistry, Burapha University Muang Chonburi 20131 Thailand; Department of Biochemistry, Faculty of Science, Burapha University Muang Chonburi 20131 Thailand; Department of Chemistry, Faculty of Science, Chulalongkorn University Phayathai Road, Pathumwan Bangkok 10330 Thailand; Center of Excellence in Materials and Biointerfaces, Chulalongkorn University Phayathai Road, Pathumwan Bangkok 10330 Thailand

## Abstract

A simple one-pot synthesis of biocompatible and antifouling magnetite nanoparticles (Fe_3_O_4_NPs) was developed. The process involves co-precipitation and *in situ* coating of zwitterionic copolymer poly[(methacrylic acid)-*co*-(2-methacryloyloxyethyl phosphorylcholine)] (PMAMPC). The influence of one-step and two-step coating methods on the performance of modified Fe_3_O_4_NP was investigated. The PMAMPC-Fe_3_O_4_NP with a narrow particle size distribution obtained from the two-step approach were highly stable in aqueous media within a wide range of pH. The particles exhibited superparamagnetic behavior with high saturation magnetization values so that they could be easily separated from solution by a magnet. Their antifouling characteristics against 2 selected proteins, lysozyme (LYZ) and bovine serum albumin (BSA), as a function of copolymer molecular weight and composition were also evaluated. Moreover, taking advantage of having carboxyl groups in the coated copolymer, the PMAMPC-Fe_3_O_4_NP were conjugated with a model biomolecular probe, biotin. The biotin-immobilized PMAMPC-Fe_3_O_4_NP were then tested for their specific capturing of a target molecule, streptavidin. The results have demonstrated the potential of PMAMPC-Fe_3_O_4_NP prepared by the two-step *in situ* coating method for probe immobilization and subsequent biomagnetic separation of target molecules. The fact that the developed functionalizable magnetite nanoparticles are biocompatible and antifouling also opens up the possibility of their use in other biomedical-relevant applications.

## Abstract 2

A simple one-pot synthesis of biocompatible and antifouling magnetite nanoparticles (Fe_3_O_4_NPs) was developed.

## Graphical abstract

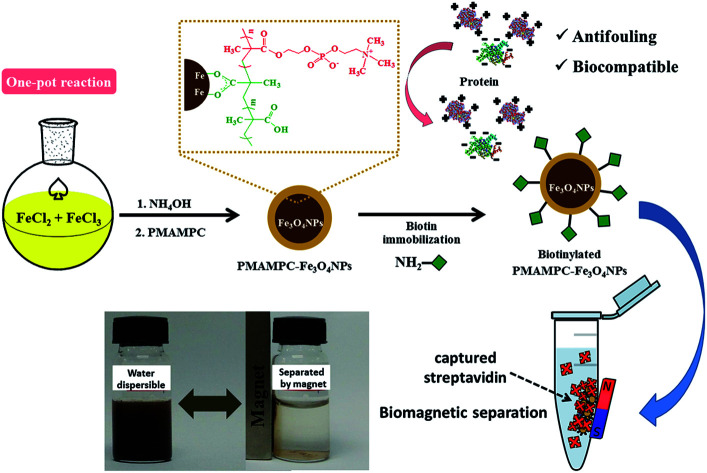

## Introduction

Superparamagnetic iron oxide nanoparticles, especially magnetite nanoparticles (Fe_3_O_4_NPs), have been widely used in a variety of biomedical applications, such as magnetic resonance imaging (MRI),^1,2^ magnetic separation,^3–5^ drug delivery,^6,7^ and biosensors.^8–10^ These applications require particles that exhibit superparamagnetic behaviour at room temperature and have high magnetization values. To be applied for biological and medical diagnosis, the magnetite nanoparticles must be stable in water at neutral pH and under physiological conditions and should not non-specifically adsorb proteins and other biological components. Coating with polymers having antifouling characteristics has been recognized as an efficient approach that can not only offer antifouling properties to but also enhance colloidal stability of the Fe_3_O_4_NPs.^11–20^ In principle, the polymer-stabilized Fe_3_O_4_NPs can be prepared by either a one-step method where *in situ* formation of the nanoparticles and the polymer coating takes place concurrently, or a two-step method where the nanoparticles are firstly formed and the polymer is then coated thereafter.

Poly(ethylene glycol) (PEG) and its derivatives have been recognized as typical hydrophilic polymers that are extensively used to modify the Fe_3_O_4_NPs for biomedical applications due to their protein resistant properties.^12–14^ Besides PEG, poly(2-methacryloyloxyethyl phosphorylcholine) (PMPC), a hydrophilic zwitterionic polymer which contains positively and negatively charged moieties within the same structure, has also received much attention as an alternative antifouling polymeric stabilizer for Fe_3_O_4_NPs.^15–20^ It has been demonstrated that PMPC can be tethered to the surface of Fe_3_O_4_NPs by surface-initiated atom transfer radical polymerization (SI-ATRP).^16–19^ This “grafting from” method required a two-step approach: surface immobilization of ATRP initiator and then PMPC formation. A simple one step *in situ* formation of PMPC-stabilized Fe_3_O_4_NPs whereby co-precipitation of ferric and ferrous salts happens simultaneously with the coating of PMPC is not possible due to a lack of anchoring group with strong affinity in the PMPC structure to bind with the surface of Fe_3_O_4_NPs. Armes and co-workers^20^ have shown that such limitation can be overcome by using a double hydrophilic block copolymer of PMPC and poly(glycerol monomethacrylate) (PGMA). PGMA would act as an absorbing block *via* a five-membered cyclic chelation between the 1,2-diol moieties in the side chain of PGMA and Fe atoms on the surface of Fe_3_O_4_NPs.^12,13^ A similar strategy has recently been reported by Zheng and co-workers,^15^ using a random copolymer of poly[(methacrylic acid)-*co*-(2-methacryloyloxyethyl phosphorylcholine)] (PMAMPC) synthesized by reversible addition fragmentation chain transfer (RAFT) polymerization. The PMAMPC-coated Fe_3_O_4_NPs were readily synthesized by coprecipitation of ferric and ferrous salts in the presence of PMAMPC and ammonium hydroxide. The carboxylate ions of the methacrylic acid (MA) units were capable of chelating to Fe atoms during the Fe_3_O_4_NPs formation, while the PMPC segment conferred colloidal stability and biocompatibility. Although one-step *in situ* coating of PMAMPC copolymer on Fe_3_O_4_NPs was successful, the values of saturation magnetization (*M*_s_) for the PMAMPC-modified Fe_3_O_4_NPs were extremely low. It was difficult to separate Fe_3_O_4_NPs having low *M*_s_ values from solution by applying a magnetic field thus centrifugation must be applied for separation.

Previously, PMPC-based copolymers have been introduced by our group as antifouling and functional polymeric layer for biosensing probe conjugation.^21,22^ Thiol-terminated poly[(methacrylic acid)-*co*-(2-methacryloyloxyethyl phosphorylcholine)] (PMAMPC-SH) was tethered to gold-coated substrate prior to conjugation with biotin employing carboxyl groups from the MA units. By using surface plasmon resonance (SPR), highly efficient detection of a specific target analyte, avidin, in diluted blood plasma was successful owning to the ability to resist nonspecific protein adsorption of the PMPC units.^21^

Although the success in preparation of magnetic iron oxide nanoparticles coated with PMPC-based polymer has been continuously reported,^15–20^ there are several problems associated with the complex synthesis methodologies and the inferior magnetic properties of the synthesized nanoparticles that limit their biomedical-relevant applications. Therefore, much attention is now focused on not only the development of simple methods to incorporate the functional polymeric layer on the surface of the magnetite nanoparticles but also providing the desirable magnetic properties. Herein, we reported a simple coating method, called a two-step *in situ* coating, where the Fe_3_O_4_NPs were firstly grown by co-precipitation and then PMAMPC was directly added to anchor onto the Fe_3_O_4_NPs, of which the formation of the nanoparticles and the polymer coating can be done in a “one-pot” system. Unlike the PMAMPC-Fe_3_O_4_NPs prepared *via* the one-step *in situ* coating of PMAMPC as previously reported by others,^15^ the PMAMPC-Fe_3_O_4_NPs prepared by the two-step *in situ* coating in this work possessed a higher saturation magnetization value so that they can be easily separated under an external magnetic field without the requirement for centrifugation. To the best of our knowledge, this is the first report on comparative studies between the one-step and two-step *in situ* coating methods for the preparation of PMAMPC-Fe_3_O_4_NPs and their properties. Additionally, the application of PMAMPC-Fe_3_O_4_NPs for biospecific separation was also demonstrated. Biotin conjugated to the carboxyl groups from MA units in the copolymer coated on PMAMPC-Fe_3_O_4_NPs were specifically capable of capturing the target molecules, streptavidin, as evaluated in comparison with the non-target molecule, bovine serum albumin (BSA).

## Materials and methods

### Chemicals

Ferrous chloride tetrahydrate (FeCl_2_·4H_2_O), ferric chloride hexahydrate (FeCl_3_·6H_2_O), ammonium hydroxide solution (NH_4_OH, 28% w/v) phosphate buffer saline (PBS), lysozyme (LYS), bovine serum albumin (BSA), 1-(3-(dimethylamino) propyl)-3-ethylcarbodiimide hydrochloride (EDC), *N*-hydroxysuccinimide (NHS), and streptavidin were purchased from Sigma-Aldrich (Singapore). Amine-PEG_2_-biotin (NH_2_-biotin) was purchased from Thermo Fisher Scientific (USA). Bio-rad dye reagent concentrate for Bradford protein assay was purchased from BIO-RAD (USA). 3-(4,5-Dimethylthiazol-2-yl)-2,5-diphenyltetrazolium bromide (MTT) and Dulbecco's Modified Eagle's medium (DMEM) were purchased from Sigma Chemical (USA). Fetal bovine serum (FBS) was purchased from Invitrogen/Gibco (USA). A series of poly[(methacrylic acid)-*co*-(2-methacryloyloxyethyl phosphorylcholine)] having varied copolymer composition (PMA_*x*_MPC_*y*_) and molecular weight were synthesized by RAFT polymerization following the reported procedure.^21^ The characteristics of the synthesized copolymers are displayed in Fig. S1 and Table S1 of the ESI.†

### Synthesis of PMAMPC-Fe_3_O_4_NPs

Fe_3_O_4_NPs were prepared and coated with PMAMPC using two *in situ* methods: one-step and two-step methods. In the case of the two-step method, the Fe_3_O_4_NPs were firstly formed by chemical co-precipitation of ferrous/ferric ions in alkaline medium and then PMAMPC solution was directly added to Fe_3_O_4_NPs suspension providing a coating of PMAMPC on Fe_3_O_4_NPs through chelating bond. Typically, FeCl_3_·6H_2_O (298 mg, 1.10 mmol) and FeCl_2_·4H_2_O (109 mg, 0.55 mmol) were dissolved in 20 mL DI water at room temperature. After the salt was completely dissolved, the mixture was added to a flask under nitrogen atmosphere at 60 °C and mechanically stirred at 750 rpm for 30 min. 6 mL of aqueous ammonia solution (28% w/v) was slowly added and the solution color changed from orange to black. The colloidal mixture was continuously stirred for another hour. Subsequently, 40 mg of PMAMPC (PMA_37_MPC_63_, 54.5 kDa) corresponding to 68.8 μmol of carboxyl groups (COOH) was directly added to the black colloidal mixture. The mixture was stirred for 1 h and cooled to room temperature. The PMAMPC-Fe_3_O_4_NPs obtained were separated by a magnet and washed with DI water several times to remove unreacted chemicals. The PMAMPC-Fe_3_O_4_NPs were dispersed in distilled water and stored as aqueous colloidal suspensions. The amounts of the PMA_37_MPC_63_ in the feed solution was 10, 20 and 40 mg. Copolymers with varied composition (PMA_21_MPC_79_, PMA_37_MPC_63_ and PMA_66_MPC_34_) and molecular weight (12.0, 25.9 and 54.5 kDa) were also used for the preparation of PMAMPC-Fe_3_O_4_NPs in order to investigate the effect of copolymer composition and molecular weight on antifouling properties.

In the case of the one-step method, the PMAMPC-Fe_3_O_4_NPs were synthesized by a chemical co-precipitation of ferrous/ferric ions in the presence of PMAMPC (PMA_37_MPC_63_, 54.5 kDa) in alkaline medium. FeCl_3_·6H_2_O (298 mg, 1.10 mmol), FeCl_2_·4H_2_O (109 mg, 0.55 mmol) and PMAMPC (40 mg, 68.8 μmol of COOH) were dissolved in 20 mL DI water at room temperature. The Fe_3_O_4_NPs were formed and simultaneously chelated with the carboxylate ions in PMAMPC after the addition of 6 mL of aqueous ammonia solution (28% w/v) under nitrogen atmosphere at 60 °C. The mixture was stirred at 750 rpm for 2 h and cooled to room temperature. The PMAMPC-Fe_3_O_4_NPs obtained were separated by a magnet and washed with DI water several times. The PMAMPC-Fe_3_O_4_NP were dispersed in distilled water and stored as aqueous colloidal suspensions. Uncoated Fe_3_O_4_NPs were also synthesized by the same method in the absence of PMAMPC.

### Characterization

The morphology, size and size distribution of the Fe_3_O_4_NPs were evaluated using transmission electron microscopy (TEM, Philips TECNAI 20). Fourier transform infrared (FT-IR) spectra were recorded by PERKIN ELMER using a KBr disk method. The amount of polymer content was determined by thermogravimetric analysis (TGA, MettlerToledo TGA/SDTA 851^e^). The weight loss of the dried samples was monitored under nitrogen atmosphere at temperatures ranging from 25 to 800 °C with a heating rate of 10 °C min^−1^. The crystalline phase was studied by X-ray diffraction (XRD, Bruker AXS Model D8 Discover). The hydrodynamic diameter and zeta potential were measured by dynamic light scattering (DLS) using a Malvern Nano ZSP instrument (UK) at room temperature at a scattering angle of 90°

### Colloidal stability study

Colloidal stability of the unmodified Fe_3_O_4_NPs and PMAMPC-Fe_3_O_4_NPs suspensions (0.1 mg mL^−1^) were visually monitored in buffer solutions having pH in the range 1–11 in the absence of external magnetic field. The quantitative measure of colloidal stability by DLS has been performed.

### Protein adsorption study

To test the antifouling properties of the PMAMPC-Fe_3_O_4_NPs, LYZ and BSA were used as models for positively charged and negatively charged proteins, respectively. The protein was dissolved in PBS (10 mM, pH 7.4). 500 μL of 1 mg mL^−1^ protein solution was added to a 500 μL colloidal solution of PMAMPC-Fe_3_O_4_NPs (2 mg mL^−1^) and incubated under gentle shaking at room temperature for 30 min. Then, the particles were magnetically separated. Supernatant was collected and analyzed for the remaining protein by the Bradford protein assay. The amount of adsorbed protein on the PMAMPC-Fe_3_O_4_NPs (*q*_e_, mg g^−1^) was calculated using eqn (1).1
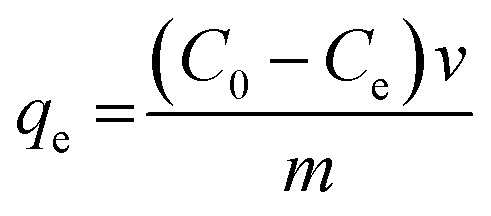
when *C*_0_ and *C*_e_ are the protein concentrations before and after adsorption (mg mL^−1^), *v* is the volume of the aqueous phase (mL), and *m* is the weight of the nanoparticles (g).

### Evaluation of cell viability in the presence of magnetite nanoparticles

An MTT assay was used to evaluate the cytotoxicity of the unmodified Fe_3_O_4_NPs and PMAMPC-Fe_3_O_4_NPs. Human umbilical vein endothelial cell line (EA.Hy926) obtained from the American-type culture collection (ATCC) were cultured in Dulbecco's Modified Eagle's Medium (DMEM) containing 10% heat-inactivated FBS. The cells were plated into a 24-well plate (1.5 × 10^5^ cells per well). After an overnight incubation, media containing various concentrations of the nanoparticles (0.0625, 0.125, 0.25 mg mL^−1^) were added to wells for 24 h. The nanoparticles were removed from the cells and then the morphology of cells before and after culturing with the nanoparticles were observed using a Olympus inverse phase contrast microscope (OLYMPUS IX70, Japan) equipped with an objective (LCAch 20X/0.04phC, Olympus, Japan) of 20× magnification. MTT solution (10 μL, 5 mg mL^−1^ in PBS) was added into each well for 2 h before aspiration of the solution. Dimethyl sulfoxide (500 μL) was added into each well to solubilize the blue formazan crystal product. Subsequently, the absorbance was measured at the wavelength of 550 nm using a microplate reader (Molecular Device, USA). The amount of formazan was proportional to the number of functional mitochondria in viable cells. All experiments were performed in triplicate and cells in culture media without the nanoparticles were used as a control. Percentage of cell viability was expressed as absorbance of treated well/absorbance of control well × 100.^23,24^ Data were expressed as means ± S.D. (*n* = 3).

### Immobilization of biotin onto PMAMPC-Fe_3_O_4_NPs

The carboxyl groups of the coated PMAMPC were first activated by an aqueous solution of EDC and NHS. A 100 μL of fresh mixed solution of EDC (50 mg mL^−1^) and NHS (50 mg mL^−1^) was added to 500 μL of aqueous suspension of PMAMPC-Fe_3_O_4_NPs (10 mg mL^−1^) for 30 min. The activated PMAMPC-Fe_3_O_4_NPs were separated from the solution by a magnet and washed with DI water. Then, the activated PMAMPC-Fe_3_O_4_NPs were re-suspended in 500 μL of NH_2_-biotin solution (10 mg mL^−1^) in DI water and incubated at room temperature overnight. After the incubation step, the nanoparticles were separated with a magnet, washed and re-suspended in DI water.

### Biospecific separation of biotinylated PMAMPC-Fe_3_O_4_NP

100 μL of biotinylated PMAMPC-Fe_3_O_4_NPs (1 mg mL^−1^) was added to 100 μL of streptavidin (1 mg mL^−1^). The mixture was mixed by vortex and incubated by shaking vigorously at room temperature for 45 min. By a magnetic separation, the supernatant solution was collected and used to determine the content of un-captured streptavidin by UV/Vis spectrophotometer. The capture efficiency (%) was calculated using eqn (2).2
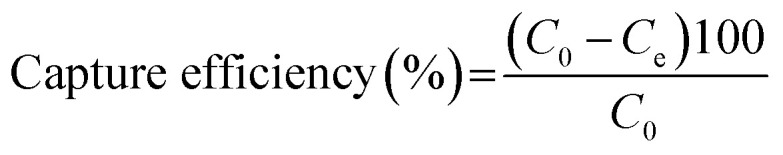
when *C*_0_ and *C*_e_ are the streptavidin concentration in solution before and after magnetic separation expressed as the absorbance value at 282 nm, respectively. A similar procedure was also performed for BSA separation.

## Results and discussion

### Synthesis and characterization of PMAMPC-coated Fe_3_O_4_NPs

**Scheme 1:**
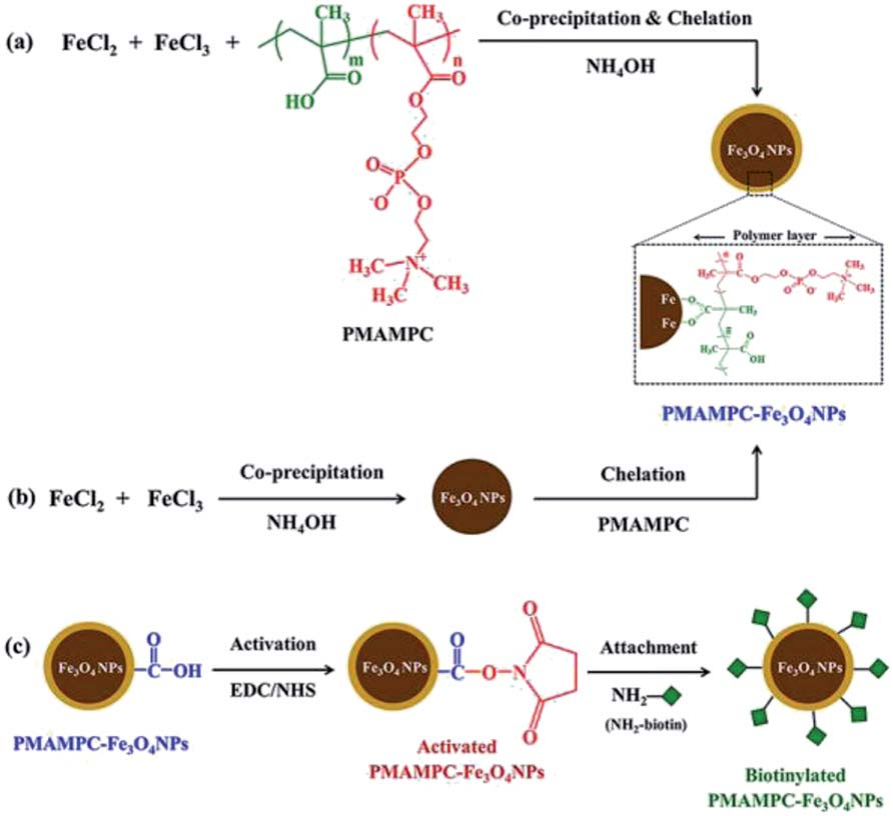
Synthesis of PMAMPC-Fe_3_O_4_NPs by (a) one-step and (b) two-step methods and (c) immobilization of NH_2_-biotin onto PMAMPC-Fe_3_O_4_NPs.

**Fig. 1:**
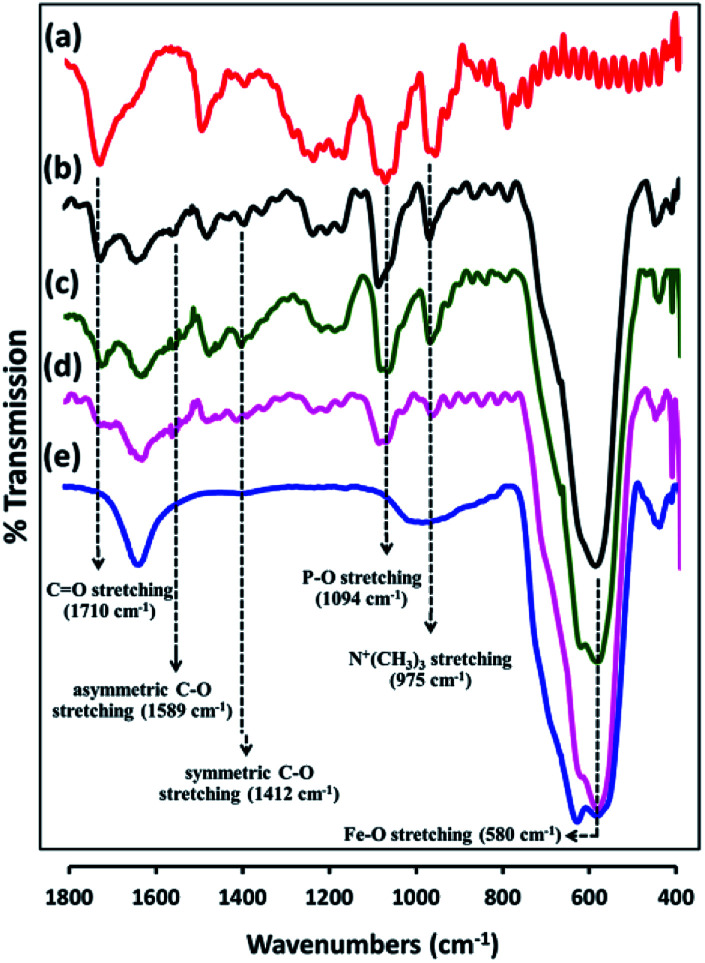
FT-IR spectra of (a) PMAMPC, PMAMPC-Fe_3_O_4_NPs synthesized by (b) two-step method, and (c) one-step method, (d) biotinylated PMAMPC-Fe_3_O_4_NPs, and (e) uncoated Fe_3_O_4_NPs.

PMAMPC-Fe_3_O_4_NPs were synthesized *via* two different *in situ* methods, the one-step and two-step methods (Scheme 1). FT-IR analysis can be used to confirm the success of PMAMPC anchoring on the Fe_3_O_4_NPs surface. As demonstrated in Fig. 1, a strong peak appearing at 580 cm^−1^ corresponded to the characteristic band of Fe–O stretching vibration mode of Fe_3_O_4_NPs. The peaks at 1630 cm^−1^ could be assigned to the stretching vibration of the hydroxyl group on the surface of the Fe_3_O_4_NPs.^25,26^ The characteristic bands of PMAMPC showed C=O, P–O and N^+^(CH_3_)_3_ stretching vibration at 1710, 1094 and 975 cm^−1^, respectively. The presence of the chelating bond was observed at 1589 cm^−1^ and 1412 cm^−1^ which can be assigned to the asymmetric and symmetric C–O stretching modes of carboxylate groups. Moreover, the wavenumber separation (*Δ*), between the asymmetric and symmetric C–O stretching of carboxylate bands can be used to distinguish the coordination types, *i.e.* monodentate, bidentate and bridging complexes.^27–30^ The large *Δ* (>200 cm^−1^) corresponds to the monodentate interaction and the small *Δ* (<110 cm^−1^) is the bidentate interaction. The medium range *Δ* (140–200 cm^−1^) was the bridging interaction. In this work, the *Δ* value is 177 cm^−1^ (1589 − 1412 = 177 cm^−1^) indicating that two Fe atoms chelated with two carboxylate oxygens, agreeing with the reported bonding between poly(acrylic acid) and Fe_3_O_4_NPs.^29,30^ From the above observations, we can confirm that PMAMPC was chemically anchored on the surface of Fe_3_O_4_NPs.

The amount of PMAMPC coated on the Fe_3_O_4_NPs can be calculated from the weight loss as determined by TGA analysis. As seen in Fig. S2 in ESI,† the uncoated Fe_3_O_4_NPs showed a major weight loss of 4% in a temperature range of 25–100 °C which should be due to loss of water. A similar magnitude of water loss from both types of PMAMPC-Fe_3_O_4_NPs was also found in the same temperature range. PMAMPC-Fe_3_O_4_NPs showed the additional weight loss between 200 and 400 °C which could be attributed to the thermal decomposition of the PMAMPC copolymer. The weight losses of 14.8 and 13.5% corresponding to 11.1% and 9.7% of PMPC content were detected for the PMAMPC-Fe_3_O_4_NPs prepared by the one-step and two-step methods, respectively. The results demonstrated that using the same amount of PMAMPC copolymer in the feed, a slightly greater PMAMPC content was incorporated into the PMAMPC-Fe_3_O_4_NPs prepared by the one-step method than that in the two-step method. This may be due to the less steric hindrance in one-step method where the incorporation of the polymer chain into the nanoparticles and the growth of the nanoparticles take place concurrently. The two-step method based on “grafting to” approach where the nanoparticles are firstly formed and the polymer is then coated on the nanoparticles surface therefore gives a low coating density since the attached polymer chains possess a steric barrier to the approaching polymer chains. So, the PMAMPC-Fe_3_O_4_NPs obtained from the two-step method should have a lower PMAMPC content.

**Fig. 2:**
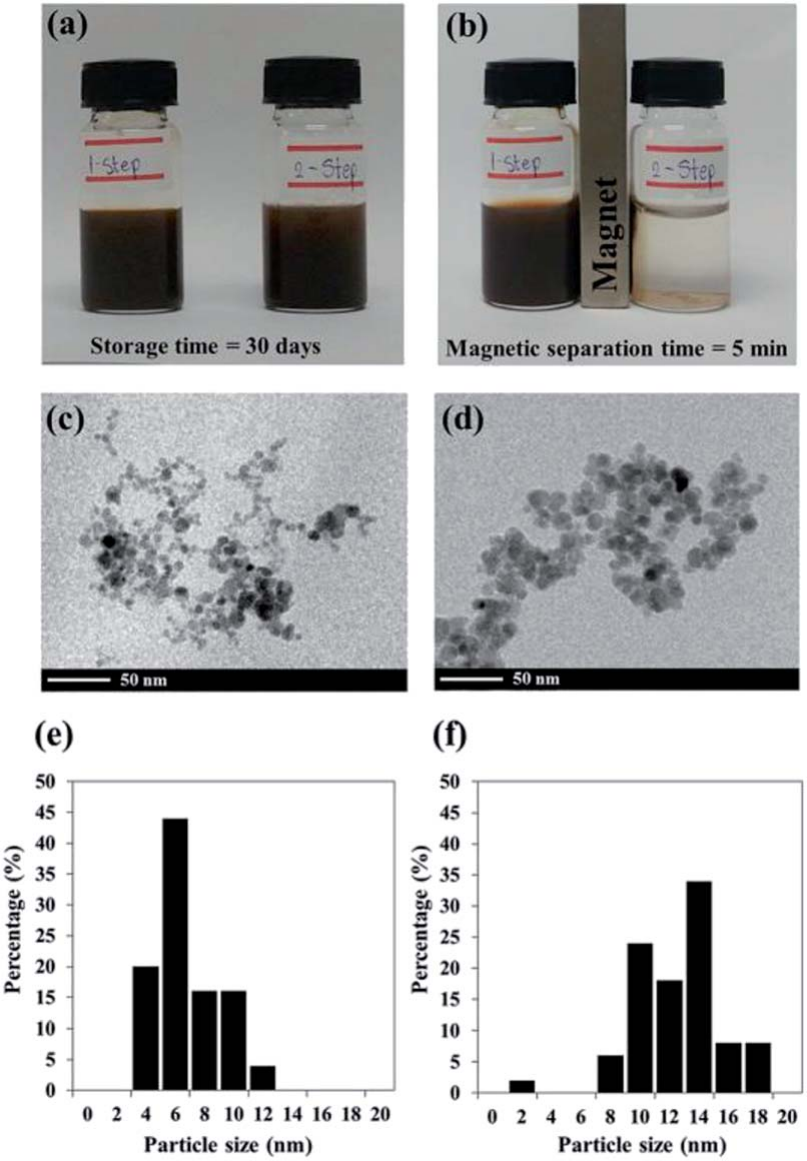
Pictures of PMAMPC-Fe_3_O_4_NPs colloidal suspensions after 30 day of storage without an external magnetic field (a) and after exposure to an external magnetic field (b), TEM images (c and d) and particle size distribution from several TEM images (*n* = 100) (e and f) of PMAMPC-Fe_3_O_4_NPs prepared by the one-step (c and e) and two-step (d and f) method.

Black colloidal suspensions of PMAMPC-Fe_3_O_4_NPs obtained from both the one-step and two-step methods showed good colloidal stability in aqueous media for up to 30 days of storage at room temperature (Fig. 2a). However, only the PMAMPC-Fe_3_O_4_NPs prepared by the two-step method could be rapidly separated from the dispersion using an external magnetic field for 5 min (Fig. 2b), showing they had an excellent magnetic response. The magnetization curves measured at room temperature for the PMAMPC-Fe_3_O_4_NPs prepared by the one-step and two-step methods are compared in Fig. S3, ESI.† There was no residual magnetism or hysteresis loop in the magnetization of both samples, suggesting the Fe_3_O_4_NPs produced are superparamagnetic. High saturation magnetization values of 48 and 47 emu g^−1^ were detected for the uncoated Fe_3_O_4_NPs and PMAMPC-Fe_3_O_4_NPs prepared by the two-step method. The saturation magnetization value of the PMAMPC-Fe_3_O_4_NPs prepared by the one-step method (38 emu g^−1^) was lower than the other samples. This may be ascribable to the lower weight fraction of the magnetic material within the magnetic nanoparticles–polymer composite as previously reported others.^15,18,30^ The magnetite content of PMAMPC-Fe_3_O_4_NPs prepared by the one-step method was given as a weight fraction of 88.9% which was lower than that of PMAMPC-Fe_3_O_4_NPs prepared by the two-step method (90.3%). The reduction of magnetization value from 47 to 38 emu g^−1^ may not seem large. However, the difference is significant enough to possess an impact on the ability to be separated by a magnet. In fact a similar observation was reported by Majeed and co-workers^31^ on the pentaerythritol tetrakis 3-mercaptopropionate-polymethacrylic acid functionalized magnetic iron oxide (PTMP-PMA-Fe_3_O_4_NP_s_). They have found that the particles cannot be separated by a magnet once the magnetization value was decreased from 58 to 45 emu g^−1^. The smaller mean particle size (7.9 ± 2.2 nm) corresponded to the PMAMPC-Fe_3_O_4_NPs prepared by the two-step method (11.7 ± 2.8 nm) (See Fig. 2c–f) may also account for the reduced magnetism.^28,29^ These results support the reasons why the PMAMPC-Fe_3_O_4_NPs prepared by the one-step method could not be separated out of the solution by a magnet.

Fig. S4 in ESI† shows the crystalline structure of uncoated Fe_3_O_4_NPs in comparison with PMAMPC-Fe_3_O_4_NPs prepared by both methods. All samples showed the standard XRD pattern of magnetite nanoparticles corresponding well with previous reports.^15,28,30^ However, the area of the diffraction peaks of the PMAMPC-Fe_3_O_4_NPs prepared by the one-step method (Fig. S4c†) were lower than those of the uncoated Fe_3_O_4_NPs and the PMAMPC-Fe_3_O_4_NPs prepared by the two-step method. The peak area data are summarized in Table S2, ESI.† It is possible that this as a consequence of the polymer coating during nanoparticle formation interfering with the crystallization process of magnetite nanoparticles. The particle sizes of the nanoparticles were also calculated using the Scherrer formula.^32^ The detail of calculation is provided in Table S3, ESI.† The sizes of PMAMPC-Fe_3_O_4_NPs prepared by the one-step and two-step method calculated from the XRD data are 9.8 and 11.4 nm, respectively which corresponded quite well with sizes evaluated by the TEM analysis (Fig. 2).

### Colloidal stability of PMAMPC-Fe_3_O_4_NPs

**Fig. 3:**
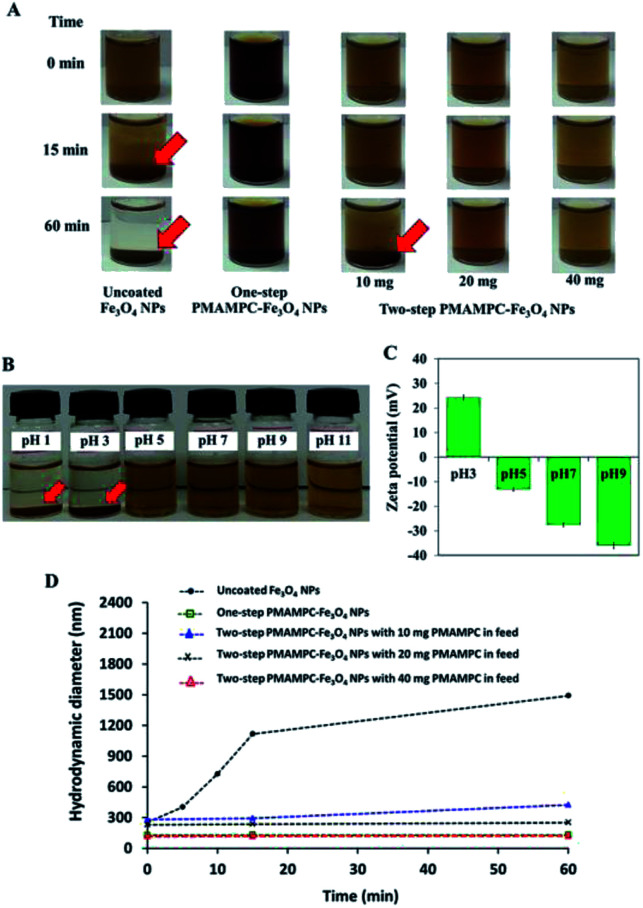
(A) Pictures of uncoated Fe_3_O_4_NPs (column 1) and PMAMPC-Fe_3_O_4_NPs colloidal suspensions prepared by one-step (column 2) and two-step methods using varied amounts of PMAMPC in the feed (column 3–5) in the absence of an external magnetic field, (B) PMAMPC-Fe_3_O_4_NPs colloidal suspensions prepared by the two-step method using 40 mg PMAMPC in the feed, (C) their corresponding zeta potential values at different pH and (D) hydrodynamic diameter of PMAMPC-Fe_3_O_4_NPs obtained from DLS.

The stability of PMAMPC-Fe_3_O_4_NPs dispersion was determined by monitoring the settling of the nanoparticles in aqueous media. As shown in Fig. 3A, the uncoated Fe_3_O_4_NPs started to aggregate and settle to the bottom of vial within 15 min (as indicated by red arrows). It could be verified with an increase in diameter of the uncoated Fe_3_O_4_NPs from nanometer range to micrometer range within 15 min as evaluated by DLS (Fig. 3D). On the other hand, the PMAMPC-Fe_3_O_4_NPs synthesized by the two *in situ* methods exhibited good colloidal stability with unchanged particle size, suggesting that the hydrophilic PMAMPC coating significantly improves the stability of Fe_3_O_4_NPs suspension. In addition, the increase of copolymer coating on Fe_3_O_4_NPs surface by increasing the PMAMPC amount in the feed solution possessed a strong impact on colloidal stability (Fig. 3A, column 3–5). The quantity of coated PMAMPC estimated from the weight loss in the temperature range 200–400 °C by TGA analysis are shown in Fig. S5, ESI.†

Because of the dissociation of COOH to COO– groups, at pH ≥ 5, the surface of PMAMPC-Fe_3_O_4_NPs would become positively charged resulting in electrostatic repulsion between neighboring nanoparticles preventing aggregation. On the other hand, the COOH groups remain protonated at pH < 5, inducing the agglomeration of nanoparticles as can be seen in Fig. 3B. The zeta potential values shown in Fig. 3D support the assumption that colloidal stability of PMAMPC-Fe_3_O_4_NPs is due to COOH dissociation.

### Protein adsorption study

**Fig. 4:**
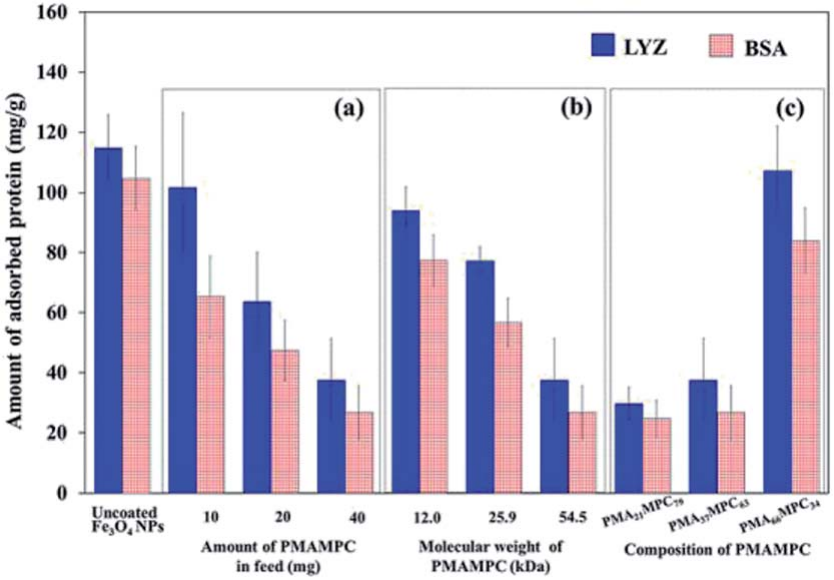
The amount of adsorbed proteins on PMAMPC-Fe_3_O_4_NPs prepared by the two-step method using (a) varied amount of PMA_37_MPC_63_ in feed, (b) varied molecular weight of PMA_37_MPC_63_ and (c) PMAMPC with varied composition.

The antifouling property of PMAMPC-Fe_3_O_4_NPs synthesized by the two-step method was determined by monitoring the adsorption of two proteins (LYS and BSA) in PBS solution (pH 7.4). As expected, the presence of PMAMPC on the surface of PMAMPC-Fe_3_O_4_NPs resulted in low adsorption of both LYS and BSA as compared with the uncoated Fe_3_O_4_NPs (Fig. 4). Under the test conditions in the PBS solution (pH 7.4, 10 mM), BSA (pI = 4.8) would be negatively charged whereas lysozyme (pI = 12) would be positively charged. The ability to resist protein adsorption is concentration-dependent. In other words, the amount of adsorbed protein decreased with increasing PMAMPC amount introduced to the feed solution during the one-pot synthesis (Fig. 4a). This coincides with the fact that the high amount of PMAMPC contained a greater number of hydrophilic MPC units on the surface of Fe_3_O_4_NPs, resulting in more effective suppression of protein adsorption. A similar result was found in both of the increasing polymer chain length and number of MPC units in the copolymer (Fig. 4b and c) in which a greater number of MPC units were obtained. The results strongly suggested that MPC units in the PMAMPC copolymer chain played an important role in preventing protein adsorption. This is in excellent agreement with many reports previously published on the fact that the introduction of MPC units in the copolymer exhibits an outstanding resistance to non-specific interactions with proteins due to its cell-membrane mimic structure.^15–22^

### Evaluation of cytotoxicity

**Fig. 5:**
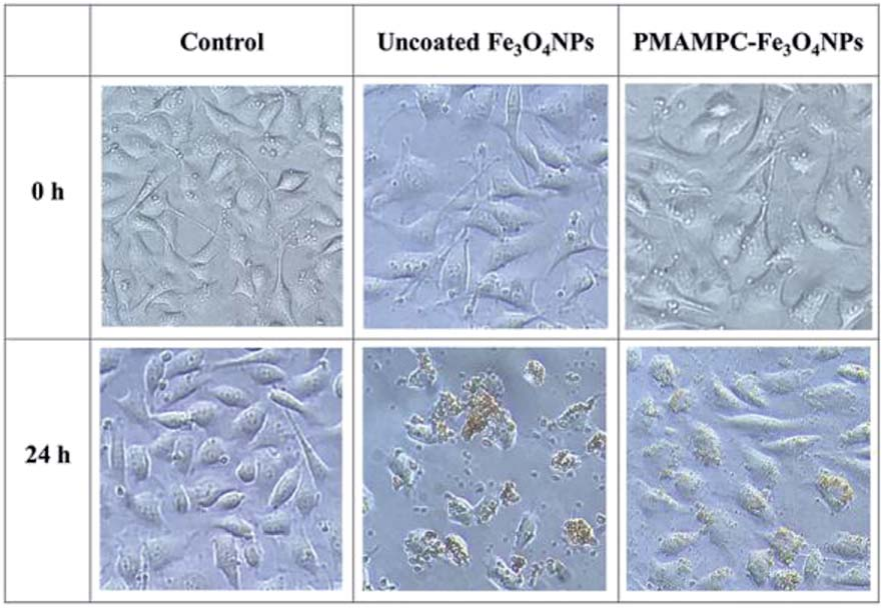
Morphology of EA.Hy926 cells before and after culturing in the absence (control) and presence of uncoated Fe_3_O_4_NPs and PMAMPC-Fe_3_O_4_NPs at 20× magnification.

**Fig. 6:**
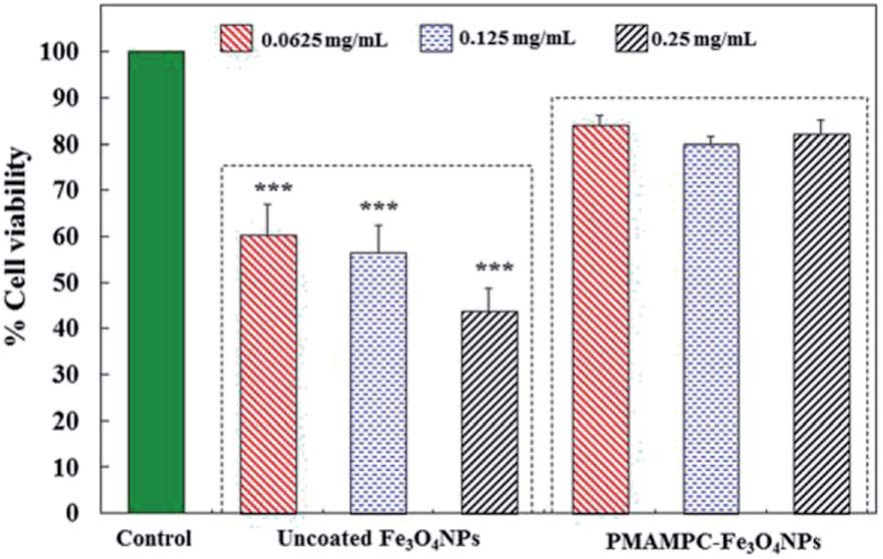
*In vitro* cytotoxicity of EA.Hy926 cells before and after culturing for 24 h in the absence (control) and presence of uncoated Fe_3_O_4_NPs and PMAMPC-Fe_3_O_4_NPs. ***p < 0.001 *vs.* control.

In this research, cytotoxicity of the Fe_3_O_4_NPs was evaluated against human umbilical vein endothelial cells (EA.Hy926). As can be seen in Fig. 5, the morphology and growth of cells were not affected by the presence of PMAMPC-Fe_3_O_4_NPs, implying that the PMAMPC-Fe_3_O_4_NPs are biocompatible and not toxic to the cells. The cell-membrane mimic structure of PMPC provides a better environment for cells so that they can maintain their activity and stability. In addition, the PMAMPC-Fe_3_O_4_NPs did not seem to attach well to the cell surface as opposed to the uncoated Fe_3_O_4_NPs which may be described as a result of the anti-fouling characteristic of PMPC-based polymer. On the other hand, cells were obviously damaged in the presence of uncoated Fe_3_O_4_NPs, indicating that they are toxic to the cells. The effect of Fe_3_O_4_NPs on the cell viability was also evaluated. The results shown in Fig. 6 indicated that PMAMPC-Fe_3_O_4_NPs slightly reduced the cell viability (80–84%) when compared with the control after culturing for 24 h. Interestingly, the cell viability remained almost unchanged when the Fe_3_O_4_NPs concentration increased, even at relatively high concentration (up to 0.25 mg mL^−1^). The uncoated Fe_3_O_4_NPs caused a significant reduction in cell viability (44–60%) in a dose-dependent manner. Apparently, the coating of zwitterionic PMAMPC significantly improves the biocompatibility of the Fe_3_O_4_NPs as evidenced by the results from cell morphology and the cell viability. This outcome is in good agreement with the work previously reported by Zheng and co-workers^15^ who have demonstrated that the PMAMPC-Fe_3_O_4_NPs prepared *via* the one-step *in situ* coating of PMAMPC were biocompatible and showed low cell uptake efficiency which correlates well with the high percentage of cell viability.

### Biomagnetic separation by PMAMPC-Fe_3_O_4_NPs

**Fig. 7:**
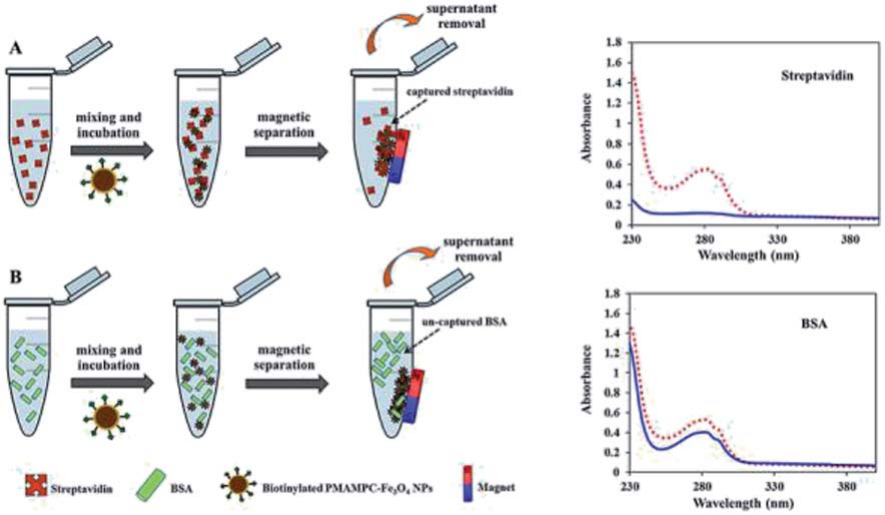
Schematic representation of the strategy for biomagnetic separation of (A) streptavidin and (B) BSA by biotinylated PMAMPC-Fe_3_O_4_NPs and their corresponding UV-vis data demonstrating the amount of protein before (red dotted line) and after (blue solid line) magnetic separation.

Besides being the anchoring points to the surface of Fe_3_O_4_NPs, the carboxyl groups of the coated PMAMPC are employed as active sites for attaching amino-functionalized biotin (NH_2_-biotin) *via* EDC/NHS coupling chemistry. The success of biotin immobilization can be verified by the decrease of the C=O stretching peak of the carboxyl groups at 1710 cm^−1^ in the FT-IR spectrum shown in Fig. 1d. The biotinylated PMAMPC-Fe_3_O_4_NPs were then further tested for specific conjugation with its target molecule, streptavidin. After the conjugation with streptavidin, separation and washing of the biotinylated PMAMPC-Fe_3_O_4_NPs can be easily performed with the aid of a magnet without the requirement for centrifugation. In order to confirm the specificity of biotinylated PMAMPC-Fe_3_O_4_NPs, the separation of streptavidin was compared with BSA, the non-target molecule (Fig. 7). In the case of streptavidin, the peak at 282 nm which corresponds to the absorbance of protein chromophores disappeared from the UV-vis spectrum after magnetic separation with biotinylated PMAMPC-Fe_3_O_4_NPs implying that the streptavidin molecules were captured and entirely removed from the solution. On the other hand, only a slight decrease in intensity of the same peak from the UV-vis spectrum of BSA was observed after magnetic separation with biotinylated PMAMPC-Fe_3_O_4_NPs. The capture efficiency can be calculated by comparing the concentration of streptavidin or BSA in solution before and after magnetic separation which were found to be 91.5% and 28.2%, respectively. The results indicate the specificity of the biotinylated PMAMPC-Fe_3_O_4_NPs for streptavidin, over BSA. The nonspecific adsorption of the biotinylated PMAMPC-Fe_3_O_4_NPs towards BSA corresponds quite well with the data displayed in Fig. 4 in which a nonspecific adsorption of BSA by the PMAMPC-Fe_3_O_4_NPs prepared from 40 mg of PMA_37_MPC_63_, 54.5 kDa was observed. This nonspecific adsorption may be suppressed if blocking of unreacted carboxyl groups is applied after biotin conjugation.

## Conclusions

A simple method for the synthesis of PMAMPC-coated Fe_3_O_4_NPs by a two-step *in situ* reaction has been demonstrated. The anchoring of PMAMPC onto the surface of Fe_3_O_4_NPs was proven to be through carboxylate chelating interactions. The PMAMPC-Fe_3_O_4_NPs showed excellent long-term colloidal stability in aqueous media at neutral pH and their stability was pH-dependent. Despite being coated by the zwitterionic copolymer, the PMAMPC-Fe_3_O_4_NPs exhibited a high saturation magnetization value so that they can be easily separated under an external magnetic field without the requirement for centrifugation. The antifouling property of PMAMPC-Fe_3_O_4_NPs was found to be dependent on the copolymer content, molecular weight and composition. The coating of zwitterionic PMAMPC can definitely improve biocompatibility of the Fe_3_O_4_NPs, suggesting that the PMAMPC-Fe_3_O_4_NPs may also be further used in contact with cells, for example, as drug carriers. The carboxyl groups in the copolymer coated on the PMAMPC-Fe_3_O_4_NPs are readily available for biotin conjugation. The biotinylated PMAMPC-Fe_3_O_4_NPs can bind specifically with the target, streptavidin as opposed to the non-specific target, BSA.

## Conflicts of interest

There are no conflicts to declare.

## Supplementary Material

RA-008-C8RA06887A-s001
