# Supplementary material for: Biocompatible zwitterionic copolymer-stabilized magnetite nanoparticles: a simple one-pot synthesis, antifouling properties and biomagnetic separation
Source: RSC Adv. 2018 Nov 5;8(65):37077–84. doi: 10.1039/c8ra06887a (PMC9089288; doi:10.1039/c8ra06887a)
Supplement: RA-008-C8RA06887A-s001 [file RA-008-C8RA06887A-s001.pdf]

Electronic Supplementary Material (ESI) for RSC Advances.
This journal is © The Royal Society of Chemistry 2018

Electronic supplementary information (ESI)

Biocompatible Zwitterionic Copolymer-stabilized Magnetite Nanoparticles: A Simple One-pot Synthesis, Antifouling Property, and Biomagnetic Separation

Supannika Boonjamnian, Thanida Trakulsujaritchok, Klaokwan Srisook, Voravee P. Hoven and Piyaporn Na Nongkhai

Page

2 The synthesis of a series of PMA_xMPC_y

3 **Fig. S1.** ^{1}H NMR spectrum for $PMA_{37}MPC_{63}$ (50 kDa)

3 **Table S1** Summary of composition and molecular weight information of a series of PMA_xMPC_y

4 **Fig. S2.** TGA curves of (a) uncoated Fe_3O_4NPs, PMAMPC-Fe_3O_4NPs synthesized by (b) two-step and (c) one-step methods.

4 **Fig. S3.** Magnetization curves of (a) uncoated Fe_3O_4NPs, PMAMPC-Fe_3O_4 NPs prepared by (b) two-step and (c) one-step methods.

5 **Fig. S4**. XRD patterns of (a) uncoated Fe_3O_4NPs, PMAMPC-Fe_3O_4NPs prepared by (b) two-step and (c) one-step methods.

5 **Table S2** Summary of peak area of uncoated Fe_3O_4NPs, PMAMPC-Fe_3O_4NPs prepared by two-step, and one-step methods.

6 The calculation of the particle size

6 **Table S3** Summary of calculated particle size of uncoated Fe_3O_4NPs and PMAMPC-Fe_3O_4NPs prepared by two-step and one-step methods from FWHM of XRD pattern.

7 **Fig. S5.** TGA curves of PMAMPC-Fe_3O_4 NPs prepared by the two-step method using varied amount of PMAMPC in feed.

The synthesis of a series of PMA$_x$MPC$_y$

MPC monomer (1.5 g, 5 mmol) was dissolved in 20 mL of mixed solvent (1:1, EtOH:PBS). After the MPC monomer was completely dissolved, MA monomer (0.43 g, 5 mmol), 4,4′-azobis(4-cyanovaleric acid) (ACVA, 18.78 mg, 0.067 mmol), and 4-cyanopentanoic acid dithiobenzoate (CPD, 55.87 mg, 0.2 mmol) were added to the monomer solution. The solution was bubbled with Ar gas for 30 min and then put in an oil bath at 70 °C for a set reaction time. The samples were withdrawn from the solution periodically to monitor the average molecular weight and dispersity (Đ) by GPC. The reaction was terminated by cooling the reaction mixture with an ice bath. The polymer solution was then purified by dialysis in DI water for 3 days and then lyophilized.

In order to vary the copolymer composition, the mole ratio of the MA and MPC monomers in the feed was varied at 30:70, 50:50 and 70:30. The ratio of monomer to CTA and the polymerization time were also varied in order to vary the molecular weight of the copolymer. The composition of the copolymer was determined by ^{1}H NMR. Fig. S1 shows a representative ^{1}H NMR spectrum of the synthesized PMAMPC. The peaks at 3.15, 0.60-1.40, and 7.4-8.2 ppm attributed to the –N(CH$_3$)$_3$ proton of the MPC unit, CH$_3$ protons of both MPC and MA units and the dithiobenzoate group at the chain end of PMAMPC. The copolymer composition can be calculated using equation below.

$$repeating\ unit\ (A) = \frac{(peak\ integration\ at\ 0.6 - 1.4\ ppm)/3}{(peak\ integration\ at\ 7.4 - 8.2\ ppm)/5} \quad \text{(eq. S1)}$$

$$MPC\ unit\ (B) = \frac{(peak\ integration\ at\ 3.15\ ppm)/9}{(peak\ integration\ at\ 7.4 - 8.2\ ppm)/5} \quad \text{(eq. S2)}$$

$$MA\ unit\ (C) = A - B \quad \text{(eq. S3)}$$

$$MPC\ composition\ (\%) = \frac{100(B)}{A} \quad \text{(eq. S3)}$$

$$MA\ composition\ (\%) = \frac{100(C)}{A} \quad \text{(eq. S4)}$$

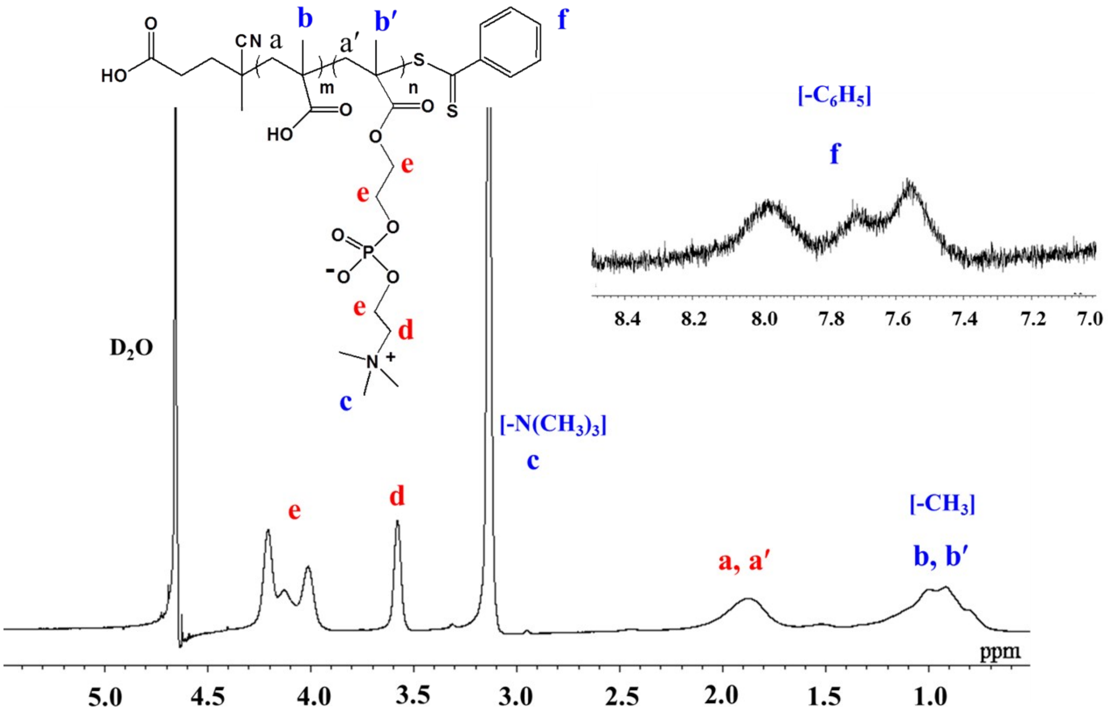

Fig. S1. ^{1}H NMR spectrum for $PMA_{37}MPC_{63}$ (50 kDa)

Table S1 Summary of composition and molecular weight information of a series of PMA_xMPC_y

Abbreviation	[monomer]:CTA	Copolymer ratio in feed (mol %)		Copolymer composition (mol %)[a]		Polymerization time (h)	$\overline{M}_n$ (x 10^3)	Đ[b]
		MA	MPC	MA	MPC			
$PMA_{21}MPC_{79}$	200	30	70	21	79	8	49.8	1.24
$PMA_{39}MPC_{61}$	50	50	50	39	61	2	12.0	1.24
$PMA_{39}MPC_{61}$	50	50	50	39	61	8	25.9	1.21
$PMA_{37}MPC_{63}$	200	50	50	37	63	8	54.5	1.23
$PMA_{66}MPC_{34}$	200	70	30	66	34	8	49.5	1.24

a = Determined from ^{1}H NMR data
b = Determined by GPC analysis

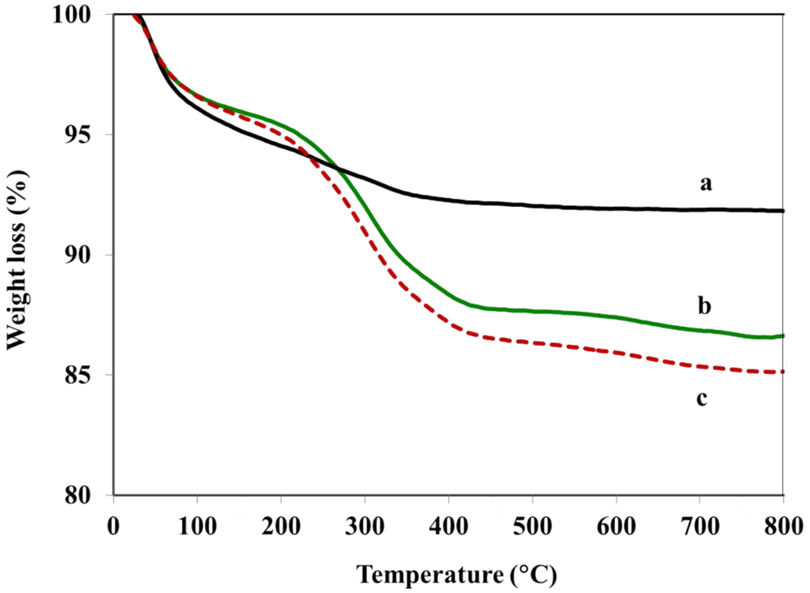

Fig. S2. TGA curves of (a) uncoated Fe_3O_4NPs, PMAMPC-Fe_3O_4NPs synthesized by (b) two-step and (c) one-step methods.

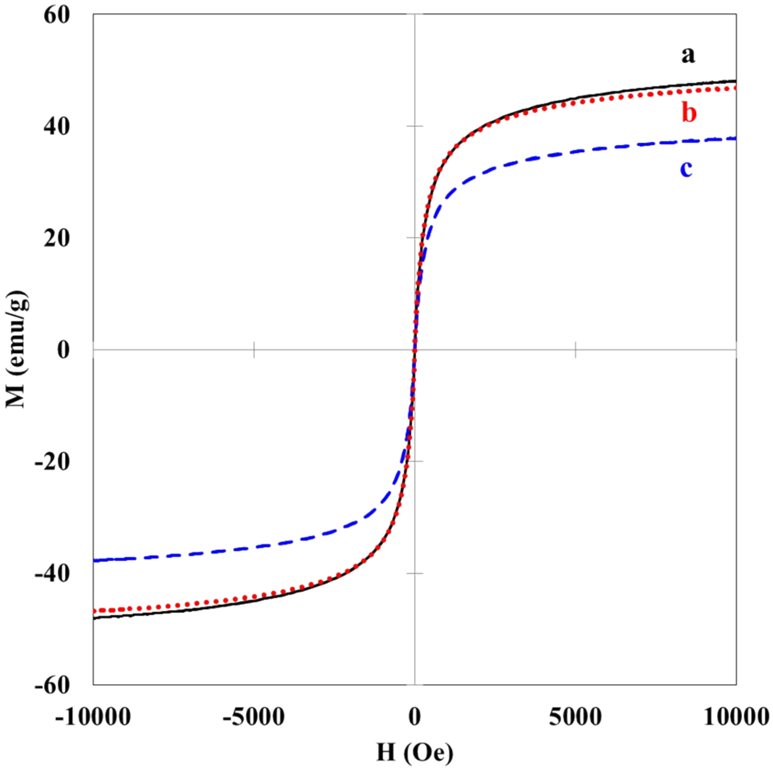

Fig. S3. Magnetization curves of (a) uncoated Fe_3O_4NPs, PMAMPC-Fe_3O_4 NPs prepared by (b) two-step and (c) one-step methods.

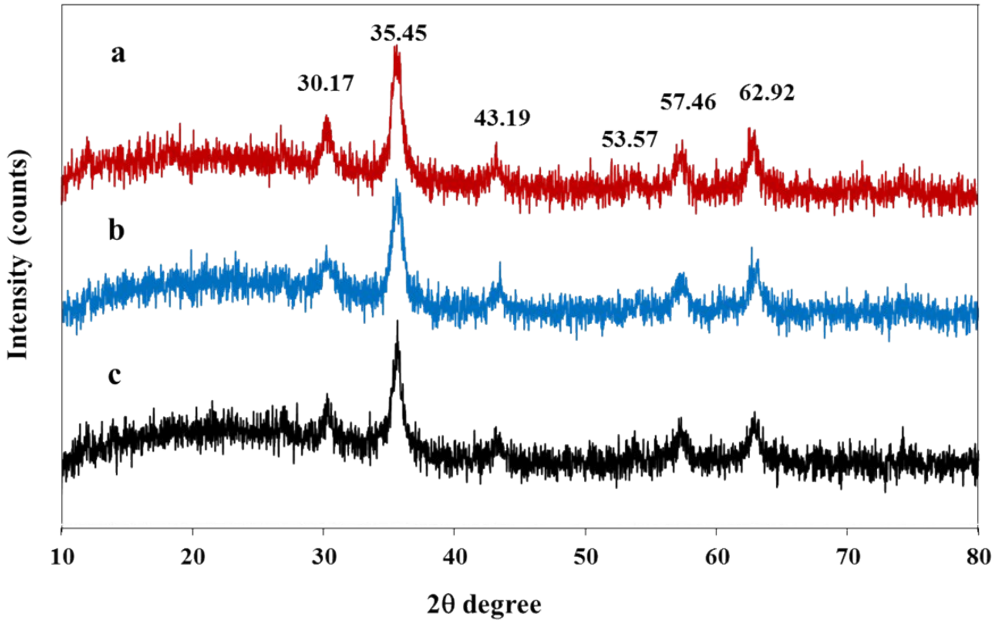

Fig. S4. XRD patterns of (a) uncoated Fe_3O_4NPs, PMAMPC-Fe_3O_4NPs prepared by (b) two-step and (c) one-step methods.

Table S2 Summary of peak area of uncoated Fe_3O_4NPs and PMAMPC-Fe_3O_4NPs prepared by two-step and one-step methods.

Samples	Peak area at different 2θ (degree)				
	30.17	35.45	43.19	57.46	62.92
Uncoated Fe_3O_4NPs	1.26	2.80	0.21	0.58	0.68
Two-step PMAMPC- Fe_3O_4NPs	1.08	2.40	0.24	0.50	0.63
One-step PMAMPC- Fe_3O_4NPs	0.90	2.27	0.18	0.45	0.43

The calculation of the particle size

The particle size of the nanoparticles was calculated with the Scherrer formula, according to the following formula:

$$D = \frac{0.89\lambda}{\beta\cos\theta} \quad \text{(eq. S5)}$$

where D is nanoparticles size (nm), λ is the wavelength of the X-ray radiation source (0.154 nm), β is full width at half maximum (FWHM) of XRD pattern and θ is Bragg's diffraction angle. The calculated values of the nanoparticles size are summarized in Table S3, ESI. The size of PMAMPC-Fe_3O_4NPs

Table S3 Summary of calculated particle size of uncoated Fe_3O_4NPs and PMAMPC-Fe_3O_4NPs prepared by two-step and one-step methods from FWHM of XRD pattern.

Samples	2θ (degree)	FWHM (radians)	Cos θ	Particles size (nm)
Uncoated Fe_3O_4NPs	35.64	0.0785	0.9520	18.33
Two-step PMAMPC- Fe_3O_4NPs	35.52	0.0126	0.9523	11.45
One-step PMAMPC- Fe_3O_4NPs	35.46	0.0148	0.9525	9.75

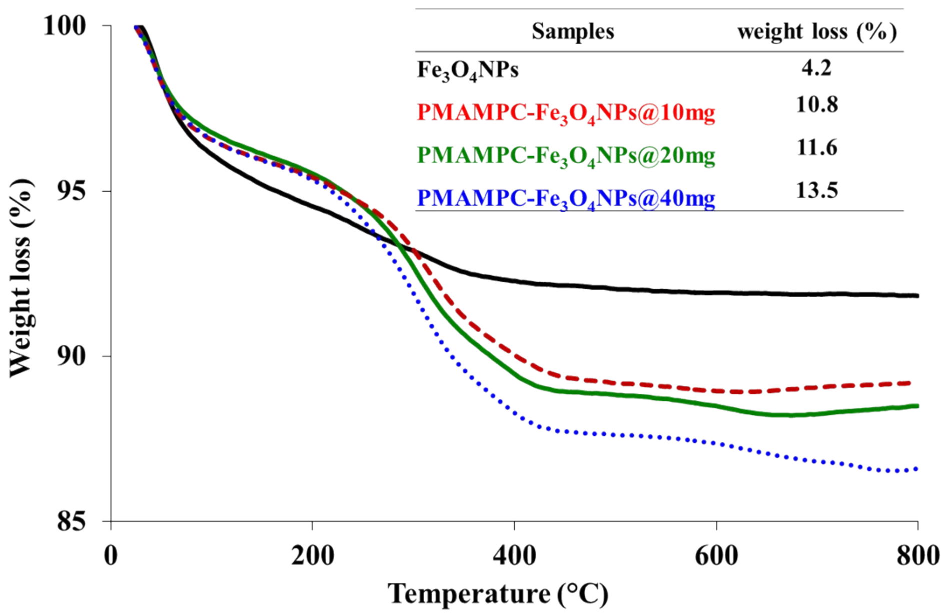

Fig. S5. TGA curves of PMAMPC-Fe_3O_4 NPs prepared by the two-step method using varied amount of PMAMPC in feed. .
